# Therapeutic Effects of Treating COVID-19 Vaccine-Induced Anti-TIF1-γ-Positive Dermatomyositis

**DOI:** 10.3390/medicina59091688

**Published:** 2023-09-20

**Authors:** Chih-Feng Wu, Wan-Ting Chen, Yen-Lin Chen, Feng-Cheng Liu

**Affiliations:** 1 School of Medicine, National Defense Medical Center, Taipei 114, Taiwan; andywu5265@gmail.com (C.-F.W.); friend1584@gapps.ndmctsgh.edu.tw (W.-T.C.); 2 Department of Psychiatry, Tri-Service General Hospital, National Defense Medical Center, Taipei 114, Taiwan; 3 Department of Pathology, Tri-Service General Hospital, National Defense Medical Center, Taipei 114, Taiwan; anthonypatho@gmail.com; 4 Division of Rheumatology, Immunology and Allergy, Department of Internal Medicine, Tri-Service General Hospital, National Defense Medical Center, Taipei 114, Taiwan

**Keywords:** COVID-19 vaccine, dermatomyositis, immunotherapy

## Abstract

An increase in skin-related autoimmune disorders has been reported as an adverse effect of coronavirus disease 2019 (COVID-19) vaccines. We present the case of a 90-year-old Taiwanese female who was newly diagnosed with anti-transcription intermediary factor 1-gamma (anti-TIF1-γ)-positive dermatomyositis (DM) after receiving a second dose of the AstraZeneca COVID-19 vaccine. Under treatment with prednisolone and monoclonal antibody therapy of abatacept, her skin lesions improved, and her muscle power increased. The serum creatinine phosphokinase level decreased from 4858 to 220 U/L, and the anti-TIF1-γ antibody titer decreased from 202 to 99. Flow cytometry data showed an increase in T cells, while NK cells, B cells (CD19), and plasma blasts all decreased. These findings suggest that standard DM treatment might be beneficial to patients with COVID-19 vaccine-induced DM.

## 1. Introduction

With the outbreak of the coronavirus disease 2019 (COVID-19), various types of vaccines have been made available for use under emergency use authorizations. However, their side effects have gradually been reported, including discomfort and itching at the site of injection, headache, and general feelings of malaise [1]. Although rare, cases of vaccine-induced dermatomyositis (DM)/polymyositis (PM) have been reported; yet the efficacy of treatment for these conditions remains uncertain [2]. Here, we present a case of a 90-year-old Taiwanese female who was newly diagnosed with DM after receiving a COVID-19 vaccine and report the response to treatment using prednisolone and abatacept.

## 2. Case Report

**Figure 1:**
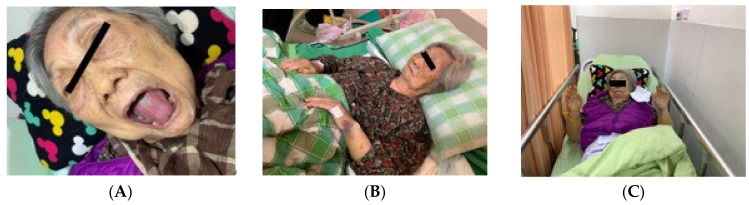
(**A**) Skin rashes over bilateral eye sockets; (**B**) skin rashes over left wrist; (**C**) proximal muscle power decreases.

**Figure 2:**
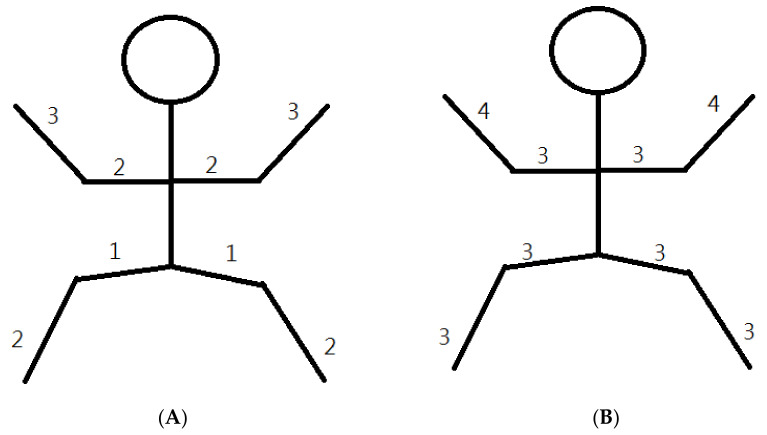
(**A**) Initially, muscle power in both proximal upper extremities scored 2, while distal strength scored 3. In both proximal lower extremities, the score was 1, with a score of 2 for the distal regions. (**B**) After treatment, there was notable improvement in muscle power. In both proximal upper extremities, the score increased from 2 to 3; and in the distal regions, it improved from 3 to 4. Similarly, in both proximal lower extremities, the score advanced from 1 to 3, with the distal strength increasing from 2 to 3.

**Figure 3:**
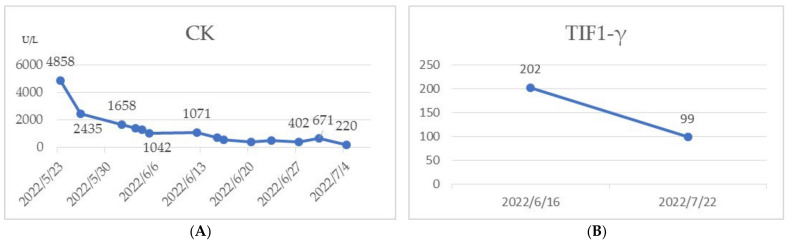
(**A**) CK level 4858 U/L initially and decreased to 220 U/L after treatment; (**B**) TIF1-γ titer before and after treatment.

**Figure 4:**
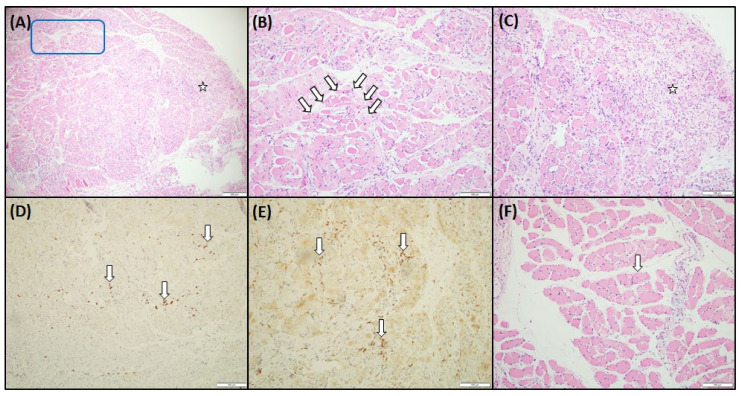
Histopathology of the muscle biopsy reveals moderate to severe muscle damage. (**A**) Low-power field showed the muscle tissue with rectangle and asterisk area for perifascicular atrophy and micro infarction. (**B**) Numerous necrotic/regenerative fibers and internal nuclei fibers are seen. Vague perifascicular atrophy (highlighted by a white arrow) is also noted. (**C**) A focus of micro infarction is found in the peripheral part of the muscle specimen (asterisk). There are only a few instances of CD8-positive (**D**) and CD4-positive (**E**) lymphocyte infiltration in the endomysial myofibers. The lymphocytes are mildly surrounding but not invading the myofibers. Some lymphocytes are also seen in the perimysial areas. (**F**) Other parts of the muscle showed milder disease involvement, and only occasionally are internal nuclei fibers seen. However, no rimmed vacuoles are seen in the specimen.

A 90-year-old female who had no previous history of rheumatology diseases or family history experienced dysphagia, severe vomiting, myalgia, rashes on her face, neck, and back, and severe mucositis 3 weeks after receiving her second AstraZeneca SARS-CoV-19 vaccine. Physical examination revealed violaceous plaques over face and anterior aspect of the neck and chest (Figure 1) and a symmetrical decrease in proximal muscle strength (Figure 2A). (Proximal upper extremities on both sides scored 2. Distal portions of both upper extremities scored 3. Proximal lower extremities on both sides scored 1. Distal areas of both lower extremities scored 2.) Blood biochemistry data revealed an elevated creatinine phosphokinase (CK) level of 4858 U/L (normal range 41–153 U/L) (Figure 3A), a C-Reactive protein level of 1.12 mg/dL (normal range < 0.8 mg/dL), an aspartate aminotransferase (AST) level of 72 U/L (normal range < 40 U/L), and a lactate dehydrogenase (LDH) level of 380 U/L (normal range 140–271 U/L). Serologic examinations were performed, revealing an anti-nuclear antibody titer of 1:80. Among the screening of dermatomyositis-specific antibodies, only anti-transcription intermediary factor 1-gamma (anti-TIF1-γ) antibody was positive (Figure 3B), while other autoantibodies including Mi-2α, Mi-2β, MDA 5, NXP, SAE 1, Ku, PM-Sol 75, PM-Sol 100, Jo-1, SRP, PL-7, PL-12, EJ, OJ, and Ro 52 were all negative. An esophageal transit study suggested impaired motility in the whole esophagus, compatible with abnormal esophageal motility of the connective tissue disorder. A nailfold capillary microscopy image revealed severe decrease in capillary density without other typical scleroderma patterns. The electromyography study suggested mild myopathy. The histopathology of muscle biopsy on the left biceps showed muscle tissue with rectangle and asterisk area for perifascicular atrophy and micro infarction, vague perifascicular atrophy, and lymphocyte infiltration in the muscle bundles, which were consistent with the diagnosis of DM (Figure 4). According to the 2017 American College of Rheumatology (ACR) classification criteria for idiopathic inflammatory myopathies (IIM), the patient’s score is 11.4 points, surpassing the threshold of 8.7 points. This score supports the classification of the patient as DM (Table 1) [3]. A diagnosis of dermatomyositis post COVID-19 vaccination was suspected.

**Figure 5:**
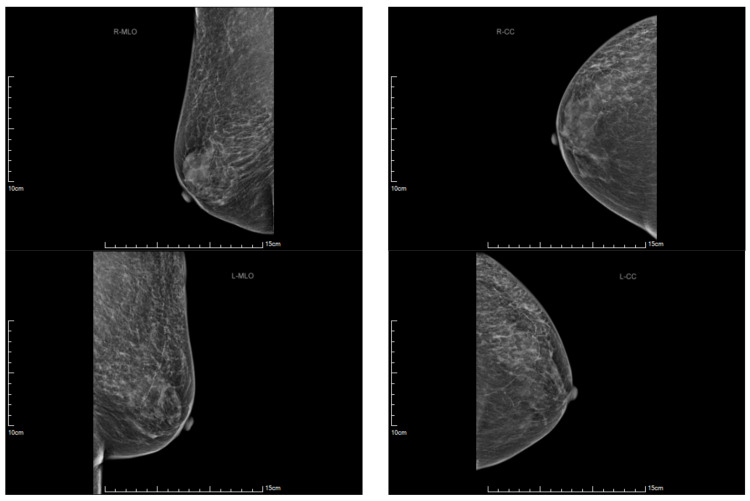
Mammography images suggest no malignancy.

To comprehensively evaluate the possibility of malignancy, we conducted extensive examinations. The results of tumor marker assessments revealed normal levels for multiple markers: Alpha-fetoprotein at 1.62 ng/mL (normal range 0–10 ng/mL), CA125 at 18.82 U/mL (normal range 0–35 U/mL), CA153 at 11.73 U/mL (normal range 0–30 U/mL), CA19-9 at 18.85 U/mL (normal range 0–37 U/mL), and carcinoembryonic antigen at 2.42 ng/mL normal range 0–5 ng/mL). Furthermore, a series of diagnostic procedures were performed, including mammography (Figure 5), gastroscopy, colonoscopy, vaginal pap smear, abdominal ultrasonography, and chest computed tomography. None of these investigations revealed evidence of malignancy. 

**Figure 6:**
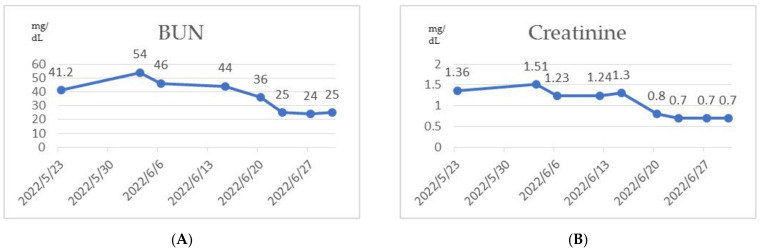
(**A**) Elevated BUN level from 41.2 to 54 mg/dL; (**B**) creatinine level from 1.36 to 1.51 mg/dL before and after treatment.

**Figure 7:**
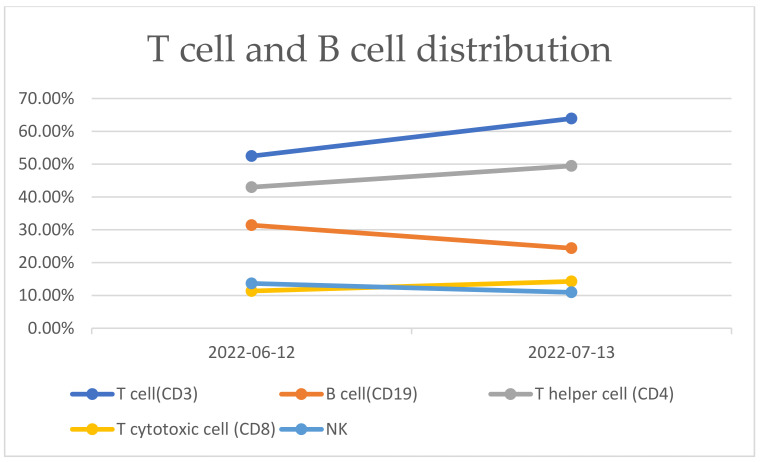
Percentage of T cells (CD3), T helper cells (CD4), and T cytotoxic cells (CD8) were all increased after treatment. Percentage of NK cells and B cells (CD19) were decreased after treatment.

After receiving pulse corticosteroid therapy 30 mg and monoclonal antibody abatacept 500 mg (Day 1), her violaceous plaques diminished, and symptoms of dysphagia were relieved. We shifted from intravenous to oral medication, including prednisolone 20 mg, hydroxychloroquine 400 mg, and methotrexate 7.5 mg (Day 10). After ten days of treatment, we changed medication to methylprednisolone 20 mg and leflunomide 10 mg due to poor renal function, as indicated by elevated blood urea nitrogen (BUN) levels, which increased from 41.2 to 54 mg/dL (normal range 7–25 mg/dL) (Figure 6A), and creatinine levels, which rose from 1.36 to 1.51 mg/dL (normal range 0.5–0.9 mg/dL) (Figure 6B) (Day 20). Her muscle power in both proximal upper extremities improved from 2 to 3 and increased from 3 to 4 in distal. Similarly, in both proximal lower extremities, her muscle power improved from 1 to 3, with distal strength increasing from 2 to 3 (Figure 2B). Serum CK level gradually decreased from 4858 to 220 U/L (Figure 3A). AST level gradually decreased from 72 to 22 U/L. LDH decreased from 380 to 263 U/L. The level of anti-TIF1-γ Ab decreased from 202 to 99 (Figure 3B). We also performed flow cytometry analysis to compare immune cell distribution to follow up on treatment response on Day 20 and Day 50. The flow cytometry data showed that T cells (CD3) increased from 52.46% to 63.90%; T helper cells (CD4) increased from 43% to 49.47%; T cytotoxic cells (CD8) increased from 11.35% to 14.27%; and T regulatory cells (Treg cells) increased (Figure 7). NK cells decreased from 13.66% to 10.94%; B cells (CD19) decreased from 31.41% to 24.39%; and plasma blasts and double-negative B cells all decreased. However, B regulatory cells’ level was not affected.

## 3. Discussion

Although the primary cause of DM remains uncertain, immune mechanisms play an important role in pathogenesis [4,5]. DM has been reported to be possibly triggered by vaccines, including hepatitis B virus, Bacillus Calmette–Guérin (BCG), tetanus, influenza, etc. [6]. Previous studies have suggested a correlation between DM and the mRNA COVID-19 vaccine injection [7,8,9]. However, our case suggests that a protein subunit vaccine may also induce immune-mediated DM. TIF1-γ-positive dermatomyositis is diagnosed in 13–31% of DM patients who are typically aged over 60 years, presenting as myopathy, elevated serum CK and aldolase levels, and comorbidities such as malignant diseases [10]. Nonetheless, the lack of malignancy in our specific case prompts inquiry into whether the mechanism activated in vaccine-induced DM differs from the conventional one, potentially impacting treatment effectiveness. 

In the 2022 British Society for Rheumatology guidelines, glucocorticoids play a pivotal role in attaining and preserving remission in myositis. Furthermore, cyclophosphamide, rituximab, IVIG, and abatacept are endorsed as second-line treatment options. Methotrexate, azathioprine, tacrolimus, ciclosporin, and mycophenolate mofetil should be considered for managing active myositis and ensuring long-term disease remission. Previous studies have shown that the combined prescription of prednisolone and high-dose intravenous immunoglobulin was effective in improving the general condition and muscle enzyme levels in COVID-19 vaccine-induced TIF1-γ-positive dermatomyositis with malignancy [11]. In our case, after steroid pulse therapy, the patient’s clinical manifestations and data improved. Abatacept has demonstrated notable effectiveness in reducing disease activity, marking a positive outcome in the treatment of muscular manifestations of DM [12]. Mia Rodziewicz’s work has shed light on the sustained therapeutic efficacy of abatacept in managing cutaneous and esophageal manifestations of anti-TIF1-γ-positive DM [13]. Following these recommendations, we employed corticosteroids, abatacept, methotrexate, and leflunomide in our approach. The patient had improvements on global activity, muscle power, and enzyme level, which indicated the achievement of significant enhancement [14].

**Figure 8:**
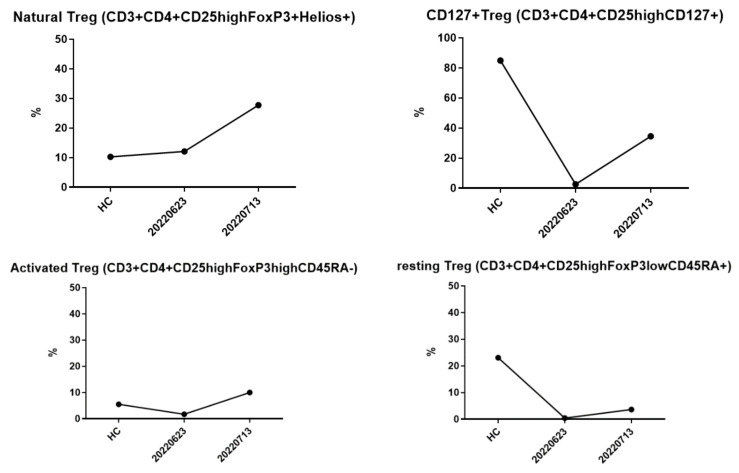
Treg cells were almost the same or even lower than the healthy control at first. After treatment, Treg cells increased after treatment.

**Figure 9:**
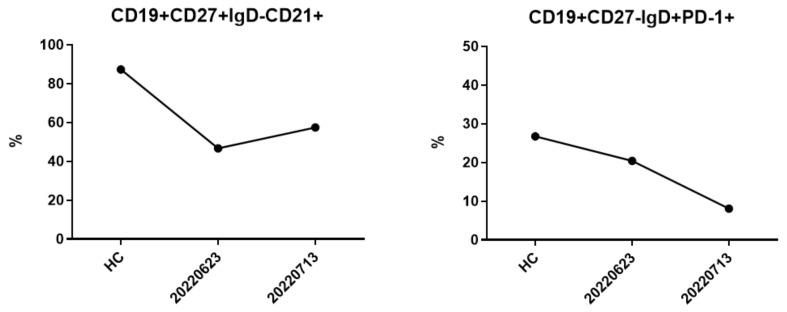

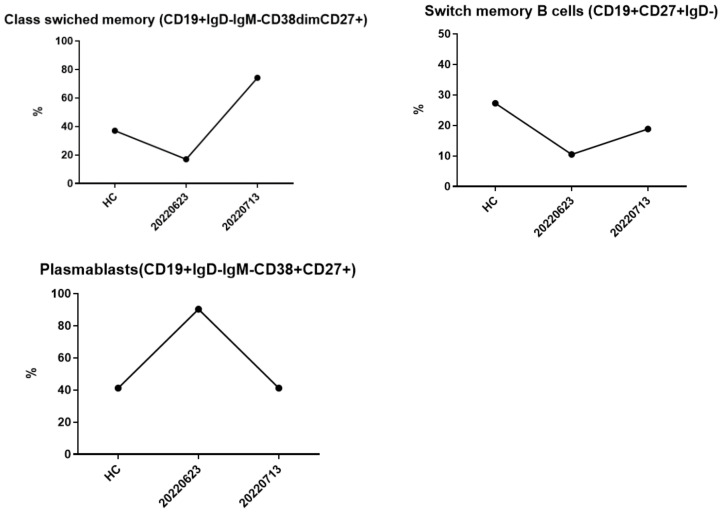
Double-negative B cells were all lower than the healthy control at first. After treatment, double-negative B cells obviously increased. Comparatively, plasma blasts were higher than the healthy people at first. After treatment, plasma blasts decreased to normal range.

**Figure 10:**
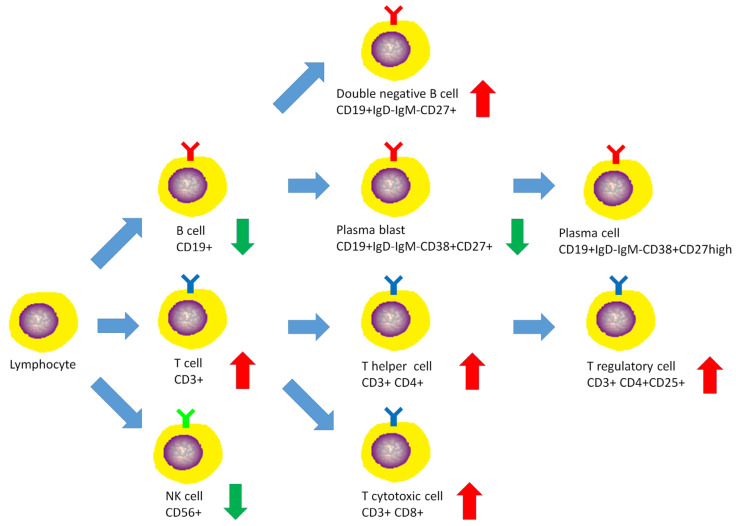
T cells, T helper cells, T cytotoxic cells, Treg cells, and double-negative B cells were all increased after pulse therapy. Otherwise, B cells, plasma blast, and NK cells were decreased after pulse therapy. (red arrow: increase, green arrow: decrease).

We further investigated the different types and percentages of immunophenotyping during our treatment course on Day 20 and Day 50. The percentages of T cells (CD3), T helper cells (CD4), and T cytotoxic cells (CD8) all increased, whereas NK cells and B cells (CD19) decreased (Figure 7). Compared to healthy controls, the percentages of natural, activated, and resting Treg cells were around the same level or even lower, but Treg cells increased significantly (Figure 8). Additionally, the level of double-negative B cells was lower than that of the healthy control at first but increased afterwards. The plasma blast level was higher than the healthy control at first but decreased to the normal range (Figure 9). The difference in immune cell distribution may be due to the use of immunomodulators. Abatacept, a type of T cell inhibitor, suppressed T cell activation, and therefore, the percentage of T cells, T helper cells, T cytotoxic cells, and Treg cells decreased initially after treatment. However, by the time we shifted treatment from Abatacept to other medication, the percentage of T cells, T helper cells, T cytotoxic cells, and Treg cells increased. Figure 10 illustrates the changes in immunophenotyping. 

## 4. Conclusions

A protein subunit COVID-19 vaccine may trigger non-malignant TIF1-γ-positive dermatomyositis. A combination of steroid and immuno therapy may be effective. 

## Figures and Tables

**Table 1:** Probability of diagnosis of dermatomyositis according to IIM classification criteria.

Classification Criteria	Yes/No	Our Patient
Age of onset of first symptom	Yes	90 years old
Objective symmetric weakness, usually progressive, of the proximal upper extremities	Yes	Muscle power in both sides’ proximal upper extremities scored 2, and distal scored 3
Objective symmetric weakness, usually progressive, of the proximal lower extremities	Yes	Muscle power in both sides’ proximal lower extremities scored 1, and distal scored 2
Neck flexors are relatively weaker than neck extensors	No	General weakness of her neck
In the legs, proximal muscles are relatively weaker than distal muscles	Yes	Muscle power in both sides’ proximal lower extremities scored 1, and distal scored 2
Heliotrope rash	No	Skin rash on her face, probable but could not accurately be defined as Heliotrope rash
Gottron’s papules	No	Probable but could not define
Gottron’s sign	No	Probable but could not define
Dysphagia or esophageal dysmotility	Yes	Dysphagia
Anti-Jo-1 (anti-Histidyl-tRNA synthetase) autoantibody positivity	No	Negative
Elevated serum levels of CK or LDH or AST or alanine aminotransferase (ALT)	Yes	CK: 4858 U/L (normal range 41–153 U/L) LDH: 380 U/L (normal range < 40 U/L) AST: 72 U/L (normal range < 40 U/L)
Endomysial infiltration of mononuclear cells surrounding, but not invading, myofibers	Yes	Muscle biopsy
Perimysial and/or perivascular infiltration of mononuclear cells	Yes	Muscle biopsy
Perifascicular atrophy	Yes	Muscle biopsy
Rimmed vacuoles	No	Absent
Score: 11.4 (>8.7), 99% probability of diagnosing as dermatomyositis		

## Data Availability

Data sharing not applicable.
